# Insulin restores renal neprilysin (NEP) and attenuates the shedding of urinary NEP and KIM-1 in diabetic Akita mice

**DOI:** 10.3389/fphar.2025.1679651

**Published:** 2026-01-12

**Authors:** Rupinder K. Gill, Esam S. B. Salem, Nadja Grobe, Khalid M. Elased

**Affiliations:** Wright State University Boonshoft School of Medicine, Dayton, OH, United States

**Keywords:** neprilysin (NEP), DKD (diabetic kidney disease), insulin, KIM-1 (kidney injury molecule 1), arginase-2 (Arg-2), urinary biomarkers of DKD, Akita diabetic mice

## Abstract

**Objective:**

Diabetic kidney disease (DKD) is characterized by dysregulation of the renin-angiotensin system (RAS) and renal tubular injury. We investigated whether insulin treatment preserves renal homeostasis by modulating neprilysin (NEP), arginase-2 (Arg-2), and kidney injury molecule-1 (KIM-1) regulation in type 1 diabetic Akita mice.

**Methods:**

Diabetic Akita mice received three subcutaneous sustained-release insulin implants (0.1 U/day) for 16 weeks. Blood measurements and urine collections were performed weekly. Western blot, enzymatic activity assays, and ELISA were used to analyze renal and urinary NEP, KIM-1, and Arg-2.

**Results:**

Full-length immunoreactive NEP (95 kDa) expression and activity were significantly reduced in Akita mice (p < 0.05 vs. wild type [WT] non-diabetic controls) in both kidney and urine. This decrease was found in both young (9-week-old) and older (27-week-old). Novel urinary immunoreactive NEP smaller fragments (70, 50, and 37 kDa) were detected in 27-week-old diabetic Akita mice but absent in non-diabetic controls mice (WT). Insulin treatment normalized hyperglycemia, reduced albuminuria, and decreased glomerular fibrosis. Furthermore, it restored renal and urinary full-length NEP expression (p < 0.05) and increased NEP activity, while reducing NEP fragment shedding. Notably, while Western blot and activity assays demonstrated reduced full-length NEP expression and activity in Akita mice, ELISA revealed a paradoxical increase in urinary NEP concentration, suggesting the detection of inactive smaller urinary NEP fragments in addition to the full-length. Urinary KIM-1 and renal Arg-2 were significantly increased in 27- weeks old diabetic Akita mice, effects that were significantly attenuated by insulin treatment (*p* < 0.05).

**Conclusion:**

Insulin therapy protects against diabetic nephropathy by: (i) augmenting renal NEP activity, (ii) reducing Arg-2-mediated injury, and (iii) attenuating tubular damage as evidenced by decreased urinary KIM-1 and NEP fragment shedding. The presence of low-molecular-weight NEP fragments in urine does warrant further investigation into their potential use as biomarkers for tracking the progression of DKD and monitoring the effectiveness of treatments.

## Introduction

Hypertension and diabetes are the main risk factors for the development of chronic kidney disease (CKD), with a substantial implication for public health worldwide. Individuals with diabetes have a 40% lifetime risk of developing diabetic kidney disease (DKD) (Alicic et al., 2017; KDIGO, 2017). CKD is a progressive loss of kidney function for more than 3 months and is mainly diagnosed by a glomerular filtration rate (GFR) below 60 mL/min/1.37 m2 and a persistent elevated albumin creatinine ratio (ACR) ≥30 mg/g (Alicic et al., 2017). DKD is the main cause of CKD, serving as the main risk factor for the development of end-stage kidney disease. Traditional screening of markers of kidney function, including estimated GFR, random ACR, and serum creatinine, reflect mainly glomerular filtration capacity but may not effectively capture early tubular injury or dysfunction. However, in the search for novel biomarkers of renal dysfunction, researchers have considered tubular injury indicators, including kidney injury molecule-1 (KIM-1), neutrophil gelatinase-associated lipocalin (NGAL), liver-type fatty acid binding protein (L-FABP), netrin-1, IL-18 and N-acetyl-β-D-glucosaminidase (NAG), which can serve as early markers of DKD (Thomas et al., 2022; Jayakumar et al., 2014; Fiseha and Tamir, 2016). The potential application of these biomarkers in the clinic has been extensively reviewed in the literature (Zeni et al., 2017; Fiseha and Tamir, 2016; Vaidya et al., 2008; Thomas et al., 2022).

The cornerstone for the management of DKD extends beyond lowering hyperglycemia, and it includes renoprotective strategies such as the use of angiotensin receptor blockers (ARBs) and angiotensin-converting enzyme inhibitors (ACE inhibitors) (Macisaac et al., 2014). The increasing prevalence of diabetes indicates the urgent need for a better understanding of the molecular mechanisms underlying DKD and the discovery of early biomarkers to help in the identification of more effective treatments. The renin-angiotensin system (RAS) is dysregulated in various pathological conditions, including DKD and is marked by elevated levels of angiotensin II (Ang II), which contributes to kidney injury via various pathogenic mechanisms, including inflammation, fibrosis, and hemodynamic disturbances (Long et al., 2004; Xu et al., 2017). Angiotensin-(1-7) [Ang-(1-7)], a protective peptide with vasodilatory, antifibrotic and anti-inflammatory properties, is primarily produced from Ang II by angiotensin converting enzyme 2 (ACE 2) (Rabelo et al., 2011; Chappell, 2016; Lin et al., 2022). Furthermore, Ang-(1-7) can also be formed by enzymatic cleavage of Ang I mainly by four enzymes, including neprilysin (NEP) (EC 3.4.24.11), prolyl-endopeptidase (EC 3.4.21.26), thimet endopeptidase (EC 3.4.24.15) (Yamamoto et al., 1992; Welches et al., 1991) and neurolysin (EC 3.4.24.16) (Rioli et al., 1998). Previous studies in our laboratory have shown increased urinary ACE2 shedding in type 2 diabetic *db*/*db* mice (Chodavarapu et al., 2013) and type 1 diabetic Akita mice (Salem et al., 2014). Furthermore, we have demonstrated that chronic hyperglycemia in *db*/*db* diabetic mice was associated with a significant reduction in renal NEP protein expression (Alawi et al., 2020). Treatment with the insulin sensitizing agent rosiglitazone effectively ameliorated hyperglycemia, restored renal NEP expression to normal levels, and markedly reduced albuminuria in these mice (Alawi et al., 2020). Furthermore, our research demonstrated that A Disintegrin and metalloprotease 17 (ADAM17) plays a crucial role in the ectodomain shedding of ACE2, resulting in increased urinary excretion of ACE2 fragments in diabetic Akita mice (Salem et al., 2014).

NEP, also known as neutral endopeptidase, CD10, enkephalinase or common acute lymphoblastic leukemia antigen (CALLA), is a zinc-dependent metalloprotease with various physiological functions (Herrmann et al., 2012). NEP is expressed in multiple organs throughout the body, including the kidney, heart, lungs, and brain, reflecting its broad physiological roles (Bayes-Genis et al., 2016). In the kidney, NEP is predominantly localized to the apical brush border membrane of proximal tubular epithelial cells where it plays a critical role in the metabolism of bioactive peptides, including those of the RAS and natriuretic peptides (Jalal et al., 1991). The structure of NEP consists of 742 amino acids and, depending on the degree of glycosylation, has a molecular weight of approximately 90–100 kDa with a short end N-terminal cytoplasmic domain and a large C-terminal extracellular catalytic domain containing the active site (Relton et al., 1983; Malfroy et al., 1988). Using immunofluorescence microscopy, our group demonstrated for the first time that renal NEP and ADAM17 co-localize in the apical brush border membrane of renal proximal tubular epithelial (Salem et al., 2014). While NEP has been identified as a primary enzyme responsible to produce renal Ang (1-7) in both mice and humans (Domenig et al., 2016), its precise role in kidney pathophysiology, particularly in the context of DKD, remains a subject of ongoing investigation. In this study, we hypothesize that diabetes-induced hyperglycemia in Akita mice leads to increased shedding of NEP fragments into the urine, which is correlated with albuminuria and the progression of DKD. To test this hypothesis, we employed a multi-faceted approach to characterize urinary NEP fragments, including *in vitro* enzyme activity assays, enzyme-linked immunosorbent assay (ELISA), and Western blot analysis. We also hypothesize that increased shedding of small fragments of urinary NEP in diabetic Akita mice is associated with hyperglycemia, albuminuria, and DKD.

Kidney injury molecule-1 (KIM-1) is a type 1 transmembrane glycoprotein that is expressed in dedifferentiated apical epithelial cells of the proximal kidney tubule (Ichimura et al., 1998; van Timmeren et al., 2007). The expression of the KIM-1 protein is virtually undetectable in healthy normal kidneys; however, it is markedly upregulated in injured renal proximal tubular epithelial cells of the human and rodent kidneys during ischemic (Ichimura et al., 1998)and toxic acute kidney injury (Ichimura et al., 2004). The metalloproteinase-dependent process causes the shedding of the soluble KIM-1 ectodomain from the cell surface into the urine (Bailly et al., 2002). Urinary KIM-1 has been recognized as a sensitive biomarker of proximal tubular injury (Vaidya et al., 2008; Nowak et al., 2016). In type 1 diabetic patients, urinary KIM-1 is associated with a lower estimated glomerular filtration rate (eGFR) and was observed in urine during progression from microalbuminuria to macroalbuminuria or from macroalbuminuria to ESRD (Panduru et al., 2015; Vaidya et al., 2011). In this investigation, our objective was to characterize KIM-1 expression in the diabetic Akita mouse model and to assess how glycemic control through insulin treatment affects KIM-1 levels, both in urine and in renal tissue.

Arginase-2 (Arg-2) has emerged as a key role in the pathogenesis of DKD and other forms of kidney injury (Morris et al., 2011). Studies have shown upregulation of renal Arg-2 protein expression in diabetic Akita mice, and treatment with the Arg-2 inhibitor effectively attenuates albuminuria and other markers of kidney damage (Morris et al., 2011). Multiple studies have highlighted the potential of inhibition of arginase-2 (Arg-2) as an attractive approach to prevent and manage DKD (You et al., 2015; Xiong et al., 2017; Morris et al., 2011; Raup-Konsavage et al., 2017). Arg-2 levels are known to be elevated in plasma from type II diabetic patients (Shatanawi and Momani, 2017). Due to the scarcity of information on urinary arginase-2 (Arg-2), our study aimed to examine its occurrence in the urine of diabetic Akita mice and explore potential changes induced by long-term insulin therapy.

The primary objective of this study was to investigate the modulation of NEP, KIM-1, and Arg-2 by chronic insulin treatment. Our particular focus on renal and urinary NEP led to the discovery that full-length NEP is constitutively shed in the urine and that insulin promotes its upregulation at the level of both expression and activity.

## Materials and methods

### Animals

Male diabetic Akita mice (C57BL/6-Ins2Akita/J, 6–7 weeks old) and wild-type non-diabetic control mice (WT) of the same age were obtained from The Jackson Laboratory (Bar Harbor, ME, United States). The mice were individually housed in cages at controlled room temperature (22 °C) with a 12-h light/dark cycle, provided free access to water and the standard mouse diet (Harlan Teklad, Madison, WI, United States). All experiments and surgical procedures performed on the animals were approved by the Wright State University Animal Care and Use Committee and granted an Animal Use Protocol (AUP) # 1056 on May 2018. Every effort was made to minimize animal pain and distress.

### Experimental design

Mice diabetic Akita mice (C57BL/6-Ins2Akita/J) (8–9 weeks old) and WT mice of the same age were assigned to three different groups. 1) Wild-type non-diabetic control mice (WT); 2) Sham-operated diabetic Akita mice; and 3) Diabetic Akita mice treated with insulin implants. Insulin was administered subcutaneously using three continuous slow-release insulin implants (LinBit for mice, Lin-Shin Canada, Toronto, ONT) as previously described (Salem et al., 2014). Insulin was released at a rate of 0.1 unit/day/implant and lasted at least 30 days. Mice were anesthetized with isoflurane and insulin implants were immersed in betadine solution and subcutaneously implanted using a 12-gauge needle under the middorsal skin. The LinBit insulin implants were prepared from a combination of insulin and micro recrystallized palmitic acid. Mice were monitored weekly for body weight, food intake, water intake, blood glucose, and urine output for 16 weeks. At the end of the study, mice were sacrificed by decapitation for blood collection, and the kidneys were removed and stored at −80 °C.

### Blood glucose measurement and urinary glucose assay

Blood glucose was measured weekly using a FreeStyle Lite blood glucose monitoring system (Abbott, CA, United States) and FreeStyle blood glucose test strips. A cut was made in the tail of the mouse to draw blood for measurement. The urinary glucose assay was performed using a glucose oxidase/peroxidase reagent kit from Sigma (St. Louis, MO, United States) as previously described (Somineni et al., 2014). Values were expressed in mg/dL.

### Urine collection

For 24 h of urine collection, mice were placed individually in metabolic cages with free access to food and water. Twenty microliters (µL) of protease inhibitor cocktail (cOmplete™ Lysis-M EDTA-free, Roche Diagnostics, Indianapolis, IN, United States) containing 2.5 mM phenylmethylsulfonyl fluoride (PMSF; Sigma-Aldrich, St. Louis, MO, United States) was added to each tube to prevent degradation of protein and peptide. The first urine sample collection was performed after 12 h and stored at 4 °C, while the second urine sample collection was performed after 24 h. Urine samples from both collections were pooled, centrifuged at 3,000 × g for 5 min at 4 °C. The supernatant was aliquoted and stored at −80 °C for future use.

### Western blot analysis

The kidneys were homogenized on ice using a lysis buffer containing a protease inhibitor cocktail (cOmplete™ Lysis-M EDTA-free buffer) containing 2.5 mM PMSF. The tissue homogenates were then centrifuged at 10,000 × g for 10 min at 4 °C to pellet cellular debris of the pellets. Kidney lysate (3–5 µg protein) and urine samples (2–20 μL, normalized to 0.5 μg creatinine) were separated on 8% SDS-PAGE gels and transferred to PVDF membranes (Millipore, MA, United States). The membranes were blocked and incubated overnight with primary antibody at 4 °C. The primary antibodies used for kidney lysate were goat anti-NEP (1:2500, Cat # AF1126, R&D Systems, Minneapolis, MN), rabbit anti-Arg-2 (1:500, Cat # HPA00063, Sigma-Aldrich, St. Louis, MO, United States). The primary antibodies used for urine were goat anti-NEP (1:1000, Cat # AF1126, R&D Systems, Minneapolis, MN), goat anti-albumin (1:1000, Cat # SC46289, Santa Cruz Biotechnology, Dallas, TX, United States) and goat anti-KIM-1 (1:1000, Cat # AF 1817, R&D Systems, Minneapolis, MN, United States). After, primary antibody incubation, membranes were probed with HRP-conjugated secondary antibodies: donkey anti-goat (1:2000, R&D Systems, Minneapolis, MN, United States) or donkey anti-rabbit (1:20,000, Jackson ImmunoResearch, West Grove, PA, United States). Protein immunoreactive bands were detected using Immobilon Western chemiluminescent HRP substrate (Millipore, MA, United States) and visualized by autoradiography using a medical film processor (Konica Minolta Medical and Graphic, Inc., Taiwan). The relative intensities of the bands were quantified using ImageJ software.

### Kidney histology and immunohistochemistry

Mice (n = 2/group) were anesthetized with ketamine/xylazine (100: 8 mg/kg) and transcardially perfused with cold PBS, followed by cold 4% paraformaldehyde. Kidneys were sent to AML Laboratories (Baltimore, MD, United States) for paraffin embedding, sectioning, periodic acid-Schiff (PAS), and Masson’s trichrome staining. The images were captured with an Olympus FV300 confocal microscope (Olympus, PA, United States) and analyzed using MetaMorph software (Molecular Devices, CA, United States).

### Urinary albumin and creatinine

The urinary albumin assay was performed using a quantitative set of mouse albumin ELISA kit (Bethyl Laboratories, Montgomery, TX, United States) according to the manufacturer’s instructions as previously described (Alawi et al., 2020). Urinary creatinine levels were measured using the Quidel MicroVue creatinine kit (San Diego, CA, United States) following the manufacturer’s instructions as previously described (Alawi et al., 2020).

### NEP activity

Renal and urinary NEP activity were assessed using a modified indirect fluorogenic assay, as previously described (Alawi et al., 2020). Urine and kidney samples equivalent to 0.5 µg of creatinine and 1 µg of protein, respectively, were incubated with assay buffer (50 mM Tris, 5 mM ZnCl_2_, 150 mM NaCl_2_ and 10 µM lisinopril) in the presence and absence of the NEP inhibitor, thiorphan (1 μM, Sigma Aldrich, St. Louis, MO, United States) for 30 min. This was followed by the addition of a NEP substrate (N-Succinyl-Ala-Ala-Phe-7-amido-4-methylcoumarin, Sigma Aldrich, St. Louis, MO, United States) and incubation for 60 min in the dark. Following this incubation, leucine aminopeptidase (0.1 mU) and phosphoramidon (0.2 mM) (both Sigma Aldrich, St. Louis, MO, United States) were added. The intensity of fluorescent signals from the reaction mixture was measured using a Synergy H1 microplate fluorescence reader (BioTek instruments, Winooski, VT) with an excitation wavelength (λex) of 390 nm and an emission wavelength (λem) of 460 nm.

### Renal and urinary NEP measurements

Urinary and renal NEP levels were measured using the mouse neprilysin DuoSet ELISA kit (Catalog # DY1126) obtained from R&D systems (Minneapolis, MN, United States) as previously described (Alawi et al., 2020). Briefly, 96-well microplates were coated with diluted capture antibody and incubated overnight at room temperature (RT). The plates were then washed with a wash buffer and blocked with reagent diluent for 1 h at RT. Standards and samples were prepared according to the kit protocol, added to the wells, and incubated for 2 h at RT. After washing, detection antibody, streptavidin-HRP, and substrate solution were added sequentially according to the kit instructions. Finally, the reaction was stopped using 2N H_2_SO_4_ and the final absorbance was read at 450 nm on a Biotek Synergy H1 microplate reader (BioTek Instruments, Winooski, VT, United States). The levels of NEP in the samples were determined using a standard curve generated with assay standards ranging from 94 to 6000 pg/mL.

### Statistics

Statistical analysis was performed using GraphPad Prism 9 software. All data were expressed as mean ± SE. Unpaired Student's t-tests were used to evaluate the difference between two groups, while one-way analysis of variance (ANOVA) was used to evaluate the difference between more than two groups. If a difference was recognized, a Bonferroni multiple comparison test was performed to see the significance. *P* value less than 0.05 was considered significant.

## Results

### Insulin treatment decreased blood and urinary glucose levels and attenuated albuminuria

**FIGURE 1:**
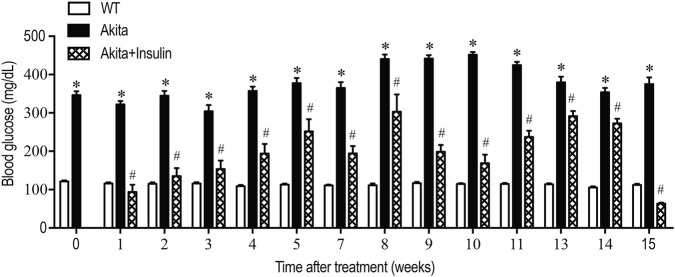
Treatment with insulin implants maintain euglycemia in diabetic Akita mice. Blood glucose levels were measured weekly over a 15-week period in wild-type nondiabetic control mice (WT); sham-operated diabetic Akita mice (Akita), and diabetic Akita mice treated with subcutaneous insulin implants (0.1 U/day) (Akita + insulin). Values are means ± SE of the group size (n = 10). Two-way ANOVA analysis followed by the Bonferroni *post hoc* test showed that insulin treatment caused a significant decrease in blood glucose levels in diabetic Akita mice (*p < 0.0001 vs. WT mice, ^#^ *p* < 0.0001 vs. diabetic Akita mice).

Blood glucose levels were monitored weekly in diabetic Akita and non-diabetic control mice (WT) from the start of the study and throughout a 16-week period following the initiation of insulin treatment. As expected, diabetic Akita mice have significantly higher blood glucose levels compared to non-diabetic control mice (WT) (Figure 1, *p* < 0.0001). Chronic insulin treatment normalized blood glucose levels within 1 week and maintained this effect throughout the study (Figure 1, *p* < 0.0001). Diabetic Akita mice exhibited a significant increase in urinary glucose compared to non-diabetic control mice (WT), which decreased significantly with insulin treatment (Table 1, *p* < 0.05). Urinary albumin excretion was measured by ELISA in 24-h urine samples collected from young (9–10 weeks) at the base line and from older (25 weeks) mice. There was a significant increase in urinary albumin excretion in diabetic Akita mice compared to non-diabetic control mice (WT) of the same age (Table 1, *p* < 0.05). However, 15 weeks of insulin treatment significantly reduced urinary albumin excretion in diabetic Akita mice compared to untreated diabetic Akita mice (Table 1, *p* < 0.05). In conclusion: insulin therapy successfully normalized diabetic indicators (blood and urinary glucose) and notably offered renal protection by decreasing heightened urinary albumin excretion in diabetic mice.

**TABLE 1:** Age-dependent changes in metabolic and urinary parameters in Wild-type, Akita, and insulin-treated diabetic Akita mice.

Parameter	WT	Akita	WT	Akita	Akita + insulin
Age (wks.)	9–10	9–10	25	25	25
Duration of treatment (wks.)	0	0	0	0	15
Group size (n)	7	10	7	8	7
Body weight (g)	21.6 ± 1.4	19.9 ± 0.9	29.5 ± 2.3	25.3 ± 1.2	27.3 ± 0.9
Urine volume (mL)	1 ± 0.2	12 ± 1.6*	1.1 ± 0.1	33.2 ± 2.4^#^	3.2 ± 0.4^$^
Blood glucose (mg/dL)	121 ± 7.8	346 ± 29.8*	112 ± 7.8	375. ± 45.7^#^	63.6 ± 5.9^$^
Urinary glucose (mg/day)	0.15 ± 0.08	65.8 ± 16.5*	0.1 ± 0.08	159.3 ± 34.17^#^	4.8 ± 3.1^$^
Urinary glucose (mg/mg creatinine)	0.56 ± 0.35	62.54 ± 7.84*	0.3 ± 0.18	94.4 ± 13.51^#^	7.3 ± 5.5^$^
Urinary albumin (µg/day)	45.5 ± 5.7	175.1 ± 9.5*	65 ± 6.0	194.1 ± 4.5^#^	55 ± 6.0^$^

Metabolic and urinary parameters were measured at the beginning of the study and over 15 weeks in wild-type non-diabetic control mice (WT); sham-operated diabetic Akita mice (Akita), and diabetic Akita mice treated with subcutaneous insulin implants (0.1 U/day) (Akita + insulin). Values represent mean ± SEM. *p < 0.05 vs. 9–10 weeks WT, ^#^ *p* < 0.05 vs. 25 weeks WT, and ^$^ *p* < 0.05 vs. 25 weeks diabetic Akita mice.

### Insulin treatment mitigated renal injury in diabetic Akita mice

**FIGURE 2:**
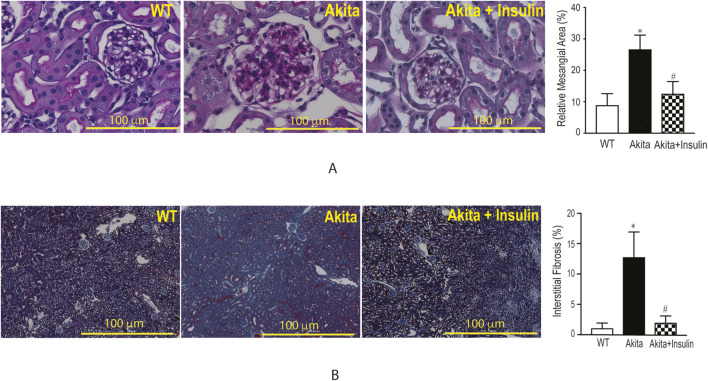
Treatment with insulin implants ameliorate diabetic nephropathy in diabetic Akita mice: **(A)** Glomerular mesangial matrix expansion: Representative photomicrographs of glomerular Periodic acid-Schiff (PAS) staining (left) and quantitative analysis of mesangial matrix expansion (right) in wild-type (WT), diabetic Akita, and insulin-treated Akita mice (n = 2 mice/group). Insulin treatment (0.1 U/day for 16 weeks) significantly attenuated the diabetes-induced increase in PAS-positive area. Values are mean ± SE (n = 15 glomeruli/kidney section). *p < 0.05 vs. WT; ^#^ *p* < 0.05 vs. untreated Akita. **(B)** Renal cortical tubulointerstitial fibrosis: Representative photomicrographs of Masson’s trichrome staining for renal cortical tubulointerstitial fibrosis (left) and corresponding quantitative analysis (right). Insulin treatment significantly reduced the diabetes-induced fibrosis. Values are mean ± SE (n = 10 non-overlapping random fields/kidney section). *p < 0.05 vs. WT; ^#^ *p* < 0.05 vs. untreated Akita.

Examination of the glomerular tufts of diabetic Akita mice revealed an increase in glomerular mesangial expansion and deposition compared to non-diabetic control mice (WT) (Figure 2A, *p* < 0.05). Quantitative analysis of periodic acid-Schiff (PAS) positive staining of the glomerular mesangial matrix in the kidney of Diabetic Akita mice demonstrated a significant decrease after 16 weeks of insulin treatment (Figure 2A, *p* < 0.05). Similarly, renal cortical tubulointerstitial fibrosis was significantly elevated in diabetic Akita mice compared to non-diabetic control mice (WT) (Figure 2B, *p* < 0.05). However, insulin treatment significantly attenuated renal fibrosis, as quantified by Masson’s trichrome staining (Figure 2B, *p* < 0.05). In conclusion, insulin therapy directly improved significant structural damage in the diabetic kidney, notably reducing both glomerular mesangial expansion and tubulointerstitial fibrosis.

### Insulin treatment increased urinary expression of the immunoreactive NEP band in diabetic Akita mice

**FIGURE 3:**
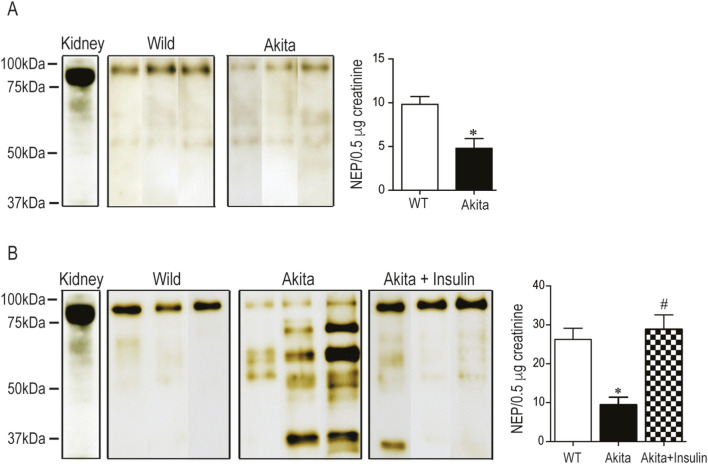
Western blot analysis reveals altered urinary NEP expression and fragmentation in diabetic Akita mice: **(A)** Western blot analysis of urinary NEP in younger (9 weeks) wild-type non-diabetic control mice (WT) and diabetic Akita mice (Akita). WT kidney lysate was used as a positive control. Immunoreactive NEP band was observed at 95 kDa for kidney lysate (first lane) and urinary samples from three WT and diabetic Akita mice. The two-sample t-test showed a significant decrease in urinary NEP expression in diabetic Akita mice compared to age-matched WT mice (*p* < 0.05). Values are means ± SE of the group size (n = 7-8). **(B)** Western blot analysis of urinary NEP in older (27 weeks) wild-type nondiabetic control mice (WT); sham operated diabetic Akita mice (Akita) and diabetic Akita mice treated with subcutaneous insulin implants (0.1 U/day) (Akita + insulin). The first line represents mouse kidney lysate (positive control). In diabetic Akita mice, smaller immunoreactive NEP fragments were observed at 70 kDa, 50 kDa, and 37 kDa. One-way ANOVA analysis of the full-length NEP band at 95 kDa revealed a significant reduction in urinary NEP expression in Akita mice compared to WT controls. Insulin treatment significantly increased urinary NEP expression in Akita mice compared to untreated Akita mice (*p < 0.05 vs. WT; ^#^ *p* < 0.05 vs. untreated diabetic Akita mice). Values are means ± SE of group sizes (n = 7–8).

The specificity of the primary anti-NEP antibody employed in this investigation was validated using NEP-knockout kidney lysates, which showed no immunoreactive bands, in contrast to the single ∼95 kDa band observed in NEP-wild-type controls (Supplementary Figure S1), corroborating the antibody’s specificity and aligning with our previous findings (Alawi et al., 2020). Western blot analysis of kidney lysates from wild-type non-diabetic control mice (WT) revealed a single, distinct immunoreactive band for NEP at approximately 95 kDa (Figure 3A), consistent with our previous studies (Alawi et al., 2020; Alawi et al., 2021). The urinary expression of the full-length immunoreactive NEP band at 95 kDa was significantly decreased in young age (9–10 weeks) diabetic Akita mice compared to age-matched non-diabetic control mice (WT) (Figure 3A, *p* < 0.05). Western blot analysis of urine samples obtained from older mice (27 weeks) revealed, for the first time, clear evidence of several small fragments of prominent immunoreactive NEP bands in diabetic Akita mice (70 kDa, 50 kDa, and 37 KDa) (Figure 3B). Like younger mice, older diabetic Akita mice showed a significant decrease in the full-length immunoreactive NEP band compared to age-matched non-diabetic control mice (WT) (Figure 3B, *p* < 0.05). During the experiment, insulin treatment normalized hyperglycemia in diabetic Akita mice for more than 15 weeks and significantly increased urinary expression of the full-length immunoreactive NEP band in diabetic Akita mice at 27 weeks (Figure 3B, *p* < 0.05). Furthermore, we demonstrate for the first time that insulin treatment in diabetic Akita mice reduces the urinary shedding of small fragments of the immunoreactive NEP bands (70 kDa, 50 kDa, and 37 KDa) (Figure 3B). In conclusion, diabetes led to a reduction in the full-length, functional NEP protein in urine and increased its fragmentation, both of which were reversed by insulin therapy, indicating NEP stabilization.

### Effect of insulin treatment on renal NEP and Arg-2 protein expression

**FIGURE 4:**
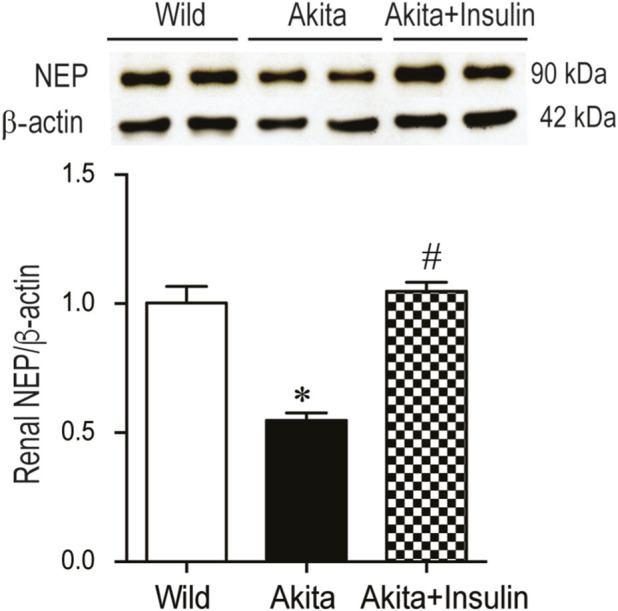
Renal NEP expression is reduced in diabetic Akita mice and restored with insulin implants treatment. Western blot analysis of renal NEP expression in kidney lysate from 27 weeks old wild-type non-diabetic control mice (WT); sham-operated diabetic Akita mice (Akita) and diabetic Akita mice treated with subcutaneous insulin implants (0.1 U/day) (Akita + insulin). An immunoreactive NEP band was observed at 95 kDa in all groups. One-way ANOVA showed a significant decrease in renal NEP expression in Akita mice compared to age-matched WT controls. Insulin treatment significantly increased renal NEP protein expression in diabetic Akita mice compared to untreated diabetic Akita mice. Values are means ± SE of the size of the group (n = 7-8). *p < 0.05 vs. WT. ^#^ *p* < 0.05 vs. untreated diabetic Akita mice.

Western blot analysis revealed a significant decrease in renal NEP protein expression in 27-week-old diabetic Akita mice compared to age-matched non-diabetic control mice (WT) (Figure 4, *p* < 0.05). After 16 weeks of insulin treatment, the renal NEP protein expression was significantly increased in diabetic Akita mice compared to untreated age-matched diabetic Akita mice (Figure 4, *p* < 0.05).

**FIGURE 5:**
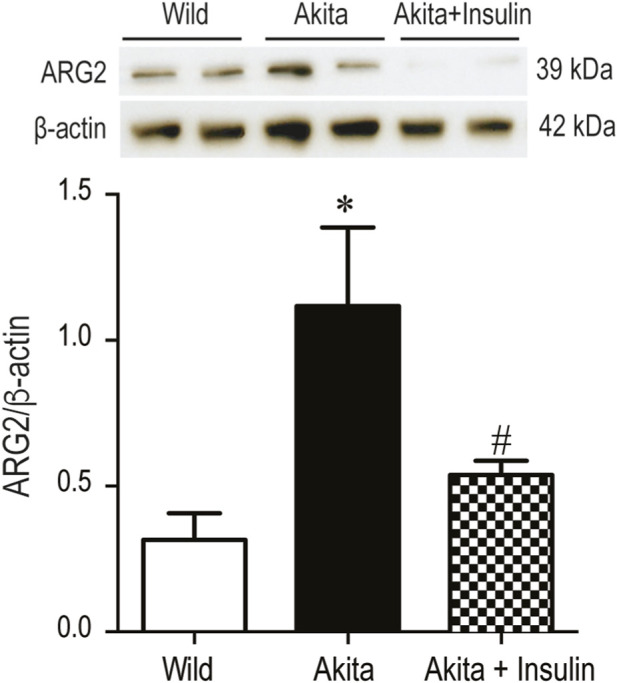
Treatment with insulin implants attenuates elevated renal Arginase-2 (Arg-2) expression in diabetic Akita mice: Western blot analysis of renal Arg-2 expression in kidney lysate from 27-week-old wild-type non-diabetic control mice (WT); sham-operated diabetic Akita mice (Akita), and diabetic Akita mice treated with subcutaneous insulin implants (0.1 U/day) (Akita + insulin). An immunoreactive Arg-2 band was detected at ∼39 kDa in all groups. One-way ANOVA showed a significant increase in renal Arg-2 expression in diabetic Akita mice compared to WT controls (*p* < 0.05). Treatment of diabetic Akita mice with insulin for 16 weeks significantly decreased renal Arg-2 expression in Akita mice compared to untreated diabetic Akita mice (*p* < 0.05). Values are means ± SE of the size of the group (n = 7-8). *p < 0.05 vs. WT. ^#^ *p* < 0.05 vs. untreated diabetic Akita mice.

Beyond exploring renal and urinary RAS, we investigated whether Arg-2 is detectable in the urine of diabetic Akia mice and whether its level could be modulated by chronic insulin treatment. Western blot analysis revealed a significant increase in renal Arg-2 expression in diabetic Akita mice compared to non-diabetic control mice (WT) (Figure 5, *p* < 0.05). Treatment of diabetic Akita mice with insulin for 16 weeks led to a significant decrease in renal Arg-2 expression compared to untreated age-matched diabetic Akita mice (Figure 5, *p* < 0.05). In conclusion, insulin therapy restored the diabetes-induced loss of renal NEP protein and decreased the diabetes-induced rise in renal Arg-2 protein, suggesting a reversal of critical molecular changes in the diabetic kidney.

### Insulin treatment increased renal and urinary NEP activity

**FIGURE 6:**
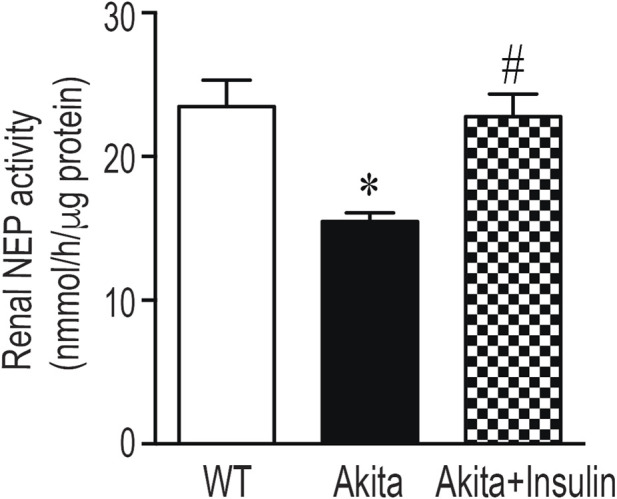
Treatment with insulin implants restores renal NEP activity in diabetic Akita mice. NEP activity was measured in kidney lysate (1 µg of protein) from 27-week-old wild-type non-diabetic control mice (WT); sham operated diabetic Akita mice (Akita) and diabetic Akita mice treated with subcutaneous insulin implants (0.1 U/day) (Akita + insulin). One-way ANOVA revealed a significant decrease in renal NEP activity in diabetic Akita mice compared to WT controls (*p* < 0.05). Insulin treatment significantly increased renal NEP activity in diabetic Akita mice compared to untreated diabetic Akita mice (*p* < 0.05). Values are means ± SE of group size (n = 7-8). *p < 0.05 vs. WT. ^#^ *p* < 0.05 vs. untreated diabetic Akita mice.

**FIGURE 7:**
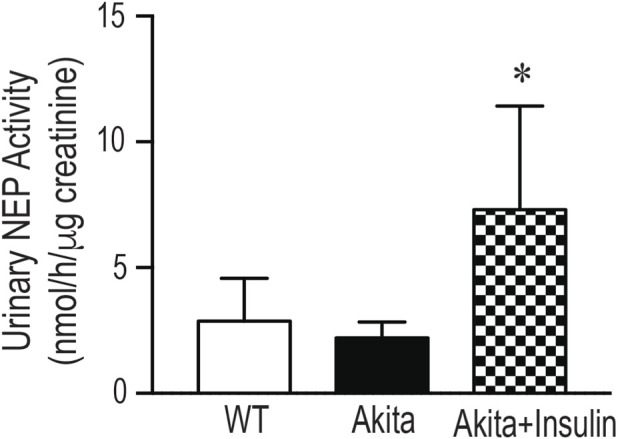
Insulin treatment enhances urinary NEP activity in diabetic Akita mice: NEP activity was determined in urine samples (normalized to 1 µg creatinine) collected from 27-week-old wild type non-diabetic control mice (WT); sham-operated diabetic Akita mice (Akita), and diabetic Akita mice treated with subcuntaneous insulin implants (0.1 U/day) (Akita + insulin). One-way ANOVA revealed no significant difference in urinary NEP activity between diabetic Akita mice and WT control mice. However, insulin treatment significantly increased urinary NEP activity in diabetic Akita mice compared to untreated diabetic Akita mice (*p* < 0.05). Values are means ± SE of group size (n = 7-8). *p < 0.05 vs. untreated Akita mice.

In agreement with Western blot data, renal NEP activity was significantly reduced in 27-week-old diabetic Akita mice compared to age-matched wild-type control mice (WT) (Figure 6, *p* < 0.05). Treatment of diabetic Akita mice with insulin for 16 weeks normalized hyperglycemia and significantly increased renal NEP activity compared to untreated age-matched diabetic Akita mice (Figure 6, *p* < 0.05). However, no significant differences in urinary NEP activity were observed between 27-weeks-old diabetic Akita mice and age-matched nondiabetic control mice (WT) (Figure 7). Paralleling the renal improvements, insulin treatment for 16 weeks significantly increased urinary NEP activity (Figure 7, *p* < 0.05). Of note, urinary NEP activity was undetectable in young (9–10 weeks) Akita mice (data not shown). In conclusion, insulin therapy restored the functional activity of the NEP enzyme in the kidney and correspondingly increased its activity in the urine, confirming the functional relevance of the observed protein level changes.

### Effect of insulin treatment on urinary NEP concentration

**FIGURE 8:**
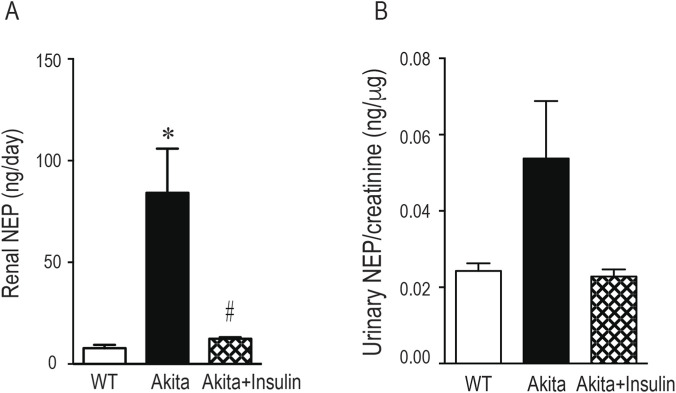
Effect of insulin treatment on ELISA quantification of urinary NEP concentration. NEP concentration in urine samples was measured using ELISA. Urine samples (20 μL, diluted 1:5) were collected from 27-week-old wild type non-diabetic control mice (WT); sham-operated diabetic Akita mice (Akita), and diabetic Akita mice treated with subcuntaneous insulin implants (0.1 U/day) (Akita + insulin). Urinary NEP concentration is presented as ng/day **(A)** and normalized to creatinine as ng/μg creatinine **(B)**. Values are means ± SE of the size of the group (n = 7-8). *p < 0.05 vs. WT. ^#^ *p* < 0.05 vs. untreated diabetic Akita mice.

Urinary NEP levels were measured in 24-h urine collections using the mouse NEP Duoset ELISA kit (R&D Systems). Interestingly, the ELISA results were inconsistent with the NEP activity assays or Western blot analysis. A key observation was the significantly elevated daily urinary NEP concentration in diabetic Akita mice compared to wild-type non-diabetic control mice (WT) (Figure 8A, *p* < 0.001). Sixteen-week insulin treatment significantly reduced daily urinary NEP excretion (ng/day) in diabetic Akita mice compared to untreated diabetic Akita mice (Figure 8A, *p* < 0.05). However, when normalized to urinary creatinine (ng/μg), no significant differences in NEP levels were observed between groups (Figure 8B). In conclusion, while ELISA showed a high NEP protein concentration in diabetic urine, this was inconsistent with other data. Notably, when normalized to creatinine, no significant differences were found, implying that the raw concentration data might be misleading.

### Effect of insulin on KIM-1 urinary shedding in diabetic Akita mice

**FIGURE 9:**
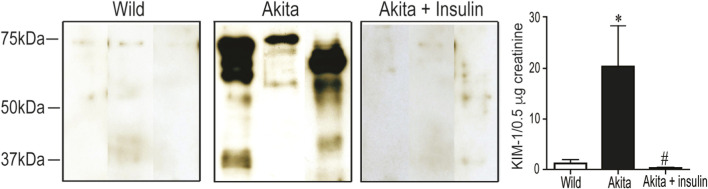
Treatment with insulin attenuates diabetes-induced urinary KIM-1 elevation in diabetic Akita mice. Western blot analysis of KIM-1 expression in urine samples (normalized to 1 µg creatinine) collected from 27 weeks old non-diabetic control mice (WT); sham-operated diabetic Akita mice (Akita), and diabetic Akita mice treated with subcutaneous insulin implants (0.1 U/day) (Akita + insulin). Lanes 1-3 represent urine samples from WT mice, lanes 4-6 represent samples from diabetic Akita mice, and lane 7-9 represent samples from Akita mice treated with insulin. An immunoreactive band for KIM-1 was observed at ∼70 kDa in urine samples from Akita mice (lanes 4–6). One-way ANOVA revealed a significant increase in urinary KIM-1 expression in diabetic Akita mice compared to WT controls (*p < 0.05). Insulin treatment significantly reduced urinary KIM-1 expression compared to untreated diabetic Akita mice (^#^ *p* < 0.05). Values are means ± SE of the size of the group (n = 7-8).

To assess renal tubular injury, we measured urinary KIM-1, a well-established biomarker, by Western blot. At 27 weeks of age, KIM-1 was undetectable in wild-type (WT) controls but was significantly elevated in diabetic Akita mice (Figure 9, *p* < 0.01). Long-term insulin treatment, which effectively normalized blood glucose for over 15 weeks, significantly attenuated this diabetes-induced KIM-1 shedding (Figure 9, *p* < 0.05). In conclusion, these findings demonstrate that increased urinary KIM-1 signifies kidney injury in diabetic Akita mice, and that insulin therapy mitigates this effect, likely through glycemic control, thereby reducing renal tubular damage in DKD.

## Discussion

This study is the first to report a significant reduction in NEP levels in kidney tissue and urine from the Akita mouse model of type 1 diabetes. In particular, the research shows that insulin administration which normalizes blood glucose levels effectively restores NEP in both kidney tissue and urine. These findings offer critical insights into the interaction between diabetes and NEP regulation, highlighting promising avenues for therapeutic intervention. Urinary NEP’s relevance is underscored by its parallels with other RAS components, such as renin, angiotensinogen, and ACE2. These are established surrogate markers for intrarenal RAS activity because their urinary concentrations reflect a balance of local synthesis, metabolism within the kidney, as well as the processes of glomerular filtration, tubular reabsorption, and excretion (Prieto et al., 2021; Lin et al., 2022). Previous studies have shown that key RAS enzymes, including ACE2 and NEP, could have renoprotective effects in models of diabetic mice (Salem et al., 2014). Although its utility as a biomarker of kidney injury has not been explored as thoroughly as that of ACE2, NGAL, or KIM-1, NEP has recently become a major focus of clinical research due to the approval of sacubitril-valsartan, a first-in-class angiotensin receptor–NEP inhibitor, for chronic heart failure with reduced ejection fraction (McMurray et al., 2014). Due to the widespread distribution of NEP in vital organs and its wide range of substrates, it plays a significant role in the pathophysiology of various diseases. There are currently no published longitudinal studies that specifically assess changes in urinary NEP levels over time in adults with diabetes. Although existing literature, including our own, demonstrates that urinary NEP is elevated in patients with type 2 diabetes and correlates with metabolic and renal parameters, these findings are limited to cross-sectional or short-term analyses and lack prospective longitudinal validation (Guillén-Gómez et al., 2018; Gutta et al., 2018). The availability of longitudinal data pertaining to serum NEP, which has been demonstrated to serve as a predictive biomarker for the future development of diabetes in adults, does not directly elucidate the dynamics of urinary NEP (Hu et al., 2023). The literature currently offers limited information on the correlation between renal NEP and diabetic kidney disease. To address this gap, we recently demonstrated a decrease in renal and urinary NEP in the *db*/*db* mouse model of type 2 diabetes (Alawi et al., 2020). The decrease in urinary NEP was effectively reversed by normalizing hyperglycemia with the PPAR-γ agonist, rosiglitazone (Alawi et al., 2020). Given that rosiglitazone affects multiple organs and acts as an insulin sensitizer, it is not clear whether correction of hyperglycemia is the only factor responsible for the upregulation of renal NEP protein expression observed in *db*/*db* mice (Alawi et al., 2020). The present study extends this work by using diabetic Akita mice. In a clinical study of patients with type 2 diabetes, systematic urinary proteome analysis using tandem mass tag labeling and bioinformatic approaches identified a significant association between increased urinary NEP and the presence of diabetic nephropathy. Specifically, NEP was among the proteins differentially expressed in the urine of patients with nephropathy, supporting its role as a candidate biomarker for diabetic kidney injury (Guillén-Gómez et al., 2018). However, interpretation of NEP changes is confounded by the concurrent use of multiple antidiabetic medications, including insulin, which may independently influence renal protein expression and urinary excretion profiles. Therefore, the direct impact of hyperglycemia on renal and urinary NEP cannot be isolated in this clinical context. Further research, including studies in animal models of diabetes, is warranted to delineate the specific effects of hyperglycemia on renal NEP expression and activity. Therefore, we considered using the mouse model of type 1 diabetes, the Akita mouse, to investigate the impact of hyperglycemia on renal and urinary NEP.

Western blot analysis of kidney tissue from diabetic Akita mice demonstrates a significant and consistent downregulation of renal NEP expression in both young (9 weeks) and older (27 weeks) animals, accompanied by a parallel reduction in renal NEP enzymatic activity. However, NEP quantification by ELISA in kidney tissue does not consistently align with either Western blot or activity assay results, likely due to the ELISA’s detection of all NEP protein forms, including inactive or fragmented species, rather than only the catalytically active enzyme. Despite the reduction in renal NEP expression and activity, urinary NEP concentrations are significantly increased in diabetic Akita mice. This paradox is explained by the fact that ELISA-based assays detect NEP fragments and inactive forms shed into the urine, which may not reflect true enzymatic activity. (Ling et al., 2024). In contrast, our selective *in vitro* NEP activity assay quantifies only the fraction of NEP that is enzymatically active, as it measures the ability of NEP to cleave a specific substrate under controlled laboratory conditions, and does not detect inactive, fragmented, or non-functional forms of the protein. The quantification of enzymatically active soluble NEP reported in this study probably corresponds to the measurement of the unaltered forms of NEP. It should be noted that a recent study, using novel epitope-directed monoclonal antibodies, demonstrated multiple circulating forms of soluble NEP (Ling et al., 2024). Therefore, measurement of enzymatically active soluble NEP provides a more accurate assessment of pathophysiological relevant NEP levels, while ELISA-based quantification may overestimate NEP due to detection of inactive fragments and degradation products. These findings highlight the importance of assay selection and interpretation when evaluating NEP as a biomarker in DKD models such as the Akita mouse.

One of the novel findings of the present study was the significantly higher urinary NEP observed in normoglycemic Akita mice after 16 weeks of insulin treatment compared to untreated hyperglycemic diabetic Akita mice. Insulin treatment in diabetic Akita mice led to an increase in urinary NEP levels, with the immunoreactive NEP band detected at approximately at 95 kDa, reflecting the profile of immunoreactive renal NEP bands as reported in our previous study (Alawi et al., 2020). Interestingly, full-length immunoreactive bands of urinary NEP were predominantly observed in non-diabetic control (WT) mice, while they were barely detectable in diabetic Akita mice. interestingly, our study revealed the presence of small fragments of immunoreactive bands for urinary NEP at 70 KDa, 50 KDa and 37 KDa in diabetic Akita mice. This prompts the suggestion that unidentified urinary enzymes may be cleaving the full-length immunoreactive NEP band in diabetic Akita mice, potentially leading to inactivation or reduction of urinary NEP activity. Considering that NEP has recognized renoprotective properties, we propose that downregulation of renal NEP could be a key factor in the development of kidney dysfunction in diabetic Akita mice. Our findings are consistent with and extend previous observations in some animal models with diabetes. For example, in streptozotocin-induced diabetic rats, a well-established model of type 1 diabetes, renal NEP levels were significantly reduced compared to non-diabetic controls (Yamaleyeva et al., 2012). This consistency between different models of diabetes strengthens the hypothesis that NEP downregulation is a common feature in DKD.

We explore the impact of normalizing hyperglycemia through insulin therapy on urinary KIM-1 levels. KIM-1 is a well-established biomarker of tubular injury, particularly sensitive to proximal tubule damage (Panduru et al., 2015). KIM-1 has gained significant recognition in the field of nephrology and drug development. In 2008, it became one of the first biomarkers qualified by the US FDA for the preclinical evaluation of acute drug-induced nephrotoxicity (Dieterle et al., 2010). Our recent research has provided valuable information on the role of KIM-1 in renal pathology. In a mouse model of renovascular hypertension using the two-kidney, one-clip (2K1C) technique, we observed a significant upregulation of KIM-1 protein expression in both renal tissue and urine samples (Alawi et al., 2021). Elevated plasma KIM-1 levels have been observed in various experimental models of kidney injury, including acute ischemia, post-reperfusion infusion, unilateral ureteral obstruction in mice, and gentamicin-induced nephrotoxicity in rats (Sabbisetti et al., 2014). A long-term study of patients with type 1 diabetes and proteinuria revealed that baseline serum KIM-1 levels were strongly associated with the rate of decline in eGFR and the risk of developing end-stage renal disease (ESRD) over a 5- to 15-year follow-up period (Sabbisetti et al., 2014). This observation underscores the potential utility of KIM-1 as a prognostic marker for chronic kidney disease progression. Although the role of KIM-1 as a urinary biomarker in various kidney diseases has been well established, its behavior in diabetic nephropathy, particularly in response to glycemic control, deserves further investigation.

In our study, we observed significantly elevated urinary KIM-1 levels in diabetic Akita mice compared to non-diabetic control (WT) mice, providing strong evidence of kidney injury in this model. Insulin administration led to a significant reduction in urinary KIM-1 levels. This suggests that glycemic control can effectively mitigate proximal tubular injury in diabetic conditions. To our knowledge, this study represents the first report of urinary KIM-1 measurements in the Akita mouse model of type 1 diabetes. These findings contribute to the growing body of evidence supporting the roles of NEP and KIM-1 in diabetic kidney disease.

Akita mice develop hyperglycemia at approximately 9 weeks of age. The hyperglycemic state persists for more than 20 weeks, allowing long-term studies. These mice show increased renal glycosuria, albuminuria, expansion of the mesangial matrix, and increased glomerular and renal fibrosis, reflecting key characteristics of human diabetic nephropathy. Insulin treatment significantly reduced both blood glucose levels and urinary glucose excretion. Albuminuria decreased after 16 weeks of insulin treatment, accompanied by reduced glomerulotubular fibrosis, suggesting improved glomerular function and/or less glomerular damage. These findings reinforce the established link between persistent hyperglycemia and the development of albuminuria, a key marker of diabetic kidney disease (Chang et al., 2012).

Recent research has highlighted the role of Arginase-2 (Arg-2) in the pathogenesis of the diabetic kidney, particularly in the diabetic Akita mice. In particular, the administration of Arg-2 inhibitors has been shown to significantly reduce albuminuria in diabetic mouse models (Morris et al., 2011). This suggests that Arg-2 plays a crucial role in the development of proteinuria, and several studies have suggested that inhibition of Arg-2 could be an attractive and appealing approach to preventing DKD (You et al., 2015; Xiong et al., 2017; Morris et al., 2011; Raup-Konsavage et al., 2017). Furthermore, our previous study revealed an upregulation of renal Arg-2 in *db*/*db* mice, a phenomenon that was subsequently downregulated by treatment with the antidiabetic drug canagliflozin (Thanekar et al., 2019). We have successfully demonstrated for the first time the presence of Arg-2 in the urine of diabetic Akita mice. Importantly, we observed that urinary Arg-2 levels were significantly altered in response to insulin therapy. This finding indicates that Arg-2 excretion is sensitive to improvements in glycemic control. This observation extends our understanding of Arg-2’s role in DKD and highlights its potential as a noninvasive biomarker. Although these results are promising, more research is needed to fully characterize the relationship between urinary Arg-2 levels, disease severity, and response to treatment in diabetic kidney disease. Future studies should aim to validate these findings in larger cohorts and explore the mechanisms underlying urinary Arg-2 excretion and modulation in diabetic conditions.

In conclusion, this study provides the first demonstration of a significant downregulation of renal NEP in diabetic Akita mice, a change associated with the development of albuminuria. Our data establish urinary NEP as a reliable indicator of intrarenal NEP levels. Importantly, we show that this dysregulation is modifiable, as insulin-mediated glycemic control directly influences NEP expression. A novel finding was the identification of small, immunoreactive NEP fragments in the urine of diabetic Akita mice. The enzymatic activity of these fragments warrants further investigation into their utility as biomarkers for tracking DKD progression and therapeutic response. Based on enzyme activity and protein expression, the renoprotective effect of insulin may be partially mediated by upregulation of renal NEP and downregulation of Arg-2. Ultimately, integrating NEP fragments into multi-biomarker panels may enhance risk stratification and personalized treatment monitoring in DKD.

## Data Availability

The raw data supporting the conclusions of this article will be made available by the authors, without undue reservation.
