# Supplementary figures and images for: Insulin restores renal neprilysin (NEP) and attenuates the shedding of urinary NEP and KIM-1 in diabetic Akita mice
Source: Front Pharmacol. 2026 Jan 12;16:1679651. doi: 10.3389/fphar.2025.1679651 (PMC12832515; doi:10.3389/fphar.2025.1679651)

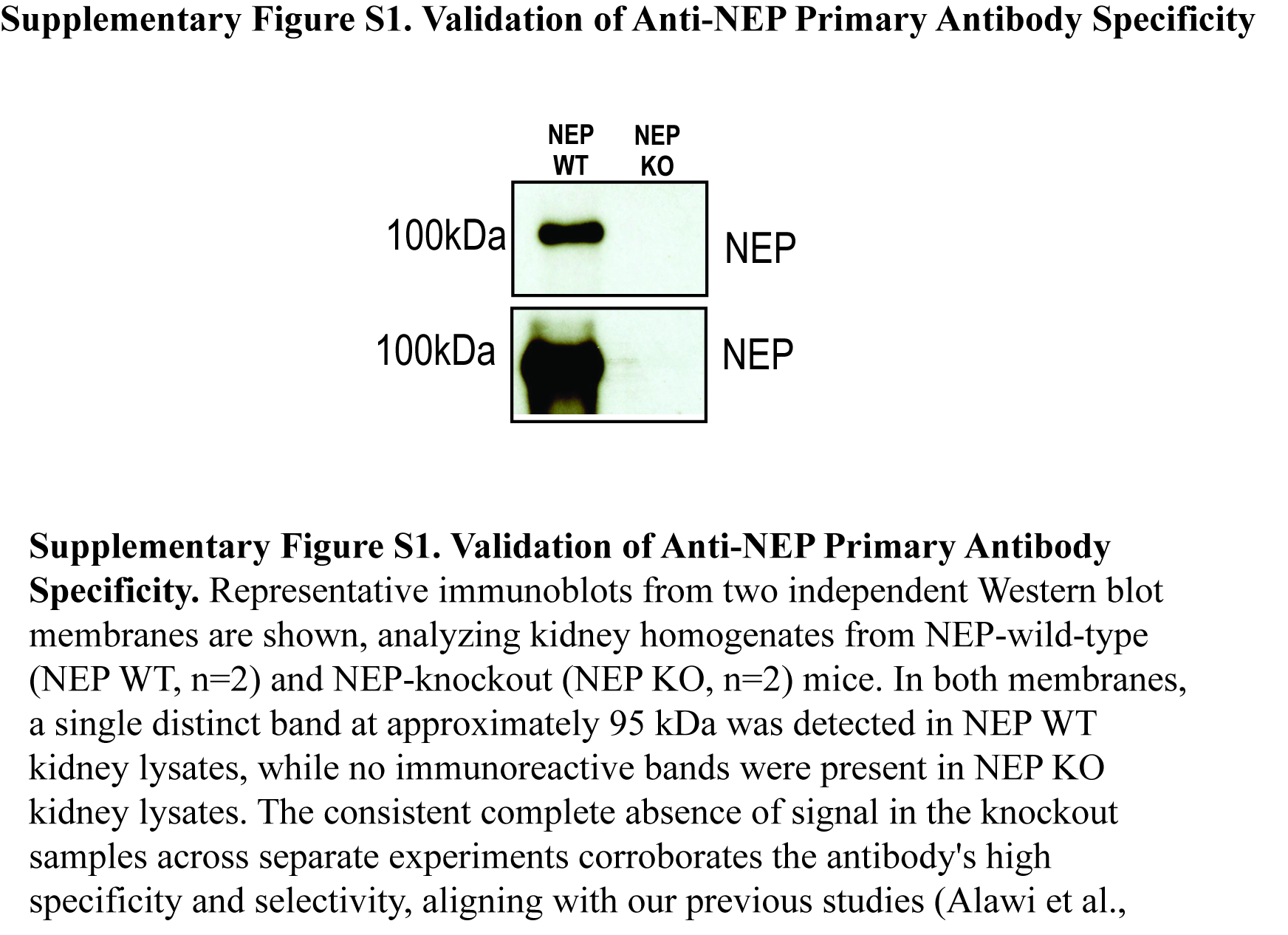

Supplement: Supplementary file 1 [file Image1.tif]
